# Epigenetic Landscape during Coronavirus Infection

**DOI:** 10.3390/pathogens6010008

**Published:** 2017-02-15

**Authors:** Alexandra Schäfer, Ralph S. Baric

**Affiliations:** Department of Epidemiology, University of North Carolina, Chapel Hill, NC 27599, USA; aschaefe@email.unc.edu

**Keywords:** coronaviruses, epigenetics, systems biology

## Abstract

Coronaviruses (CoV) comprise a large group of emerging human and animal pathogens, including the highly pathogenic severe acute respiratory syndrome coronavirus (SARS-CoV) and Middle East respiratory syndrome coronavirus (MERS-CoV) strains. The molecular mechanisms regulating emerging coronavirus pathogenesis are complex and include virus–host interactions associated with entry, replication, egress and innate immune control. Epigenetics research investigates the genetic and non-genetic factors that regulate phenotypic variation, usually caused by external and environmental factors that alter host expression patterns and performance without any change in the underlying genotype. Epigenetic modifications, such as histone modifications, DNA methylation, chromatin remodeling, and non-coding RNAs, function as important regulators that remodel host chromatin, altering host expression patterns and networks in a highly flexible manner. For most of the past two and a half decades, research has focused on the molecular mechanisms by which RNA viruses antagonize the signaling and sensing components that regulate induction of the host innate immune and antiviral defense programs upon infection. More recently, a growing body of evidence supports the hypothesis that viruses, even lytic RNA viruses that replicate in the cytoplasm, have developed intricate, highly evolved, and well-coordinated processes that are designed to regulate the host epigenome, and control host innate immune antiviral defense processes, thereby promoting robust virus replication and pathogenesis. In this article, we discuss the strategies that are used to evaluate the mechanisms by which viruses regulate the host epigenome, especially focusing on highly pathogenic respiratory RNA virus infections as a model. By combining measures of epigenome reorganization with RNA and proteomic datasets, we articulate a spatial-temporal data integration approach to identify regulatory genomic clusters and regions that play a crucial role in the host’s innate immune response, thereby defining a new viral antagonism mechanism following emerging coronavirus infection.

## 1. Coronaviruses

The severe acute respiratory syndrome coronavirus (SARS-CoV) emerged in 2002/2003, most likely in the Guangdong Province, China. From the initial outbreak, SARS-CoV rapidly spread across the globe causing 8000 infections and ~800 deaths in 28 countries; mortality rates approached 50% in aged individuals [1,2,3]. From its animal reservoir in Chinese horseshoe bats (genus *Rhinolophus*), the SARS-CoV was thought to have adapted to Palm civets and raccoon dogs in open markets, before finally colonizing human populations [4]. More recent studies have shown that SARS-CoV, as well as a large reservoir of SARS-like bat CoV (SL-CoV) have the ability to efficiently utilize the human angiotensin-converting enzyme 2 (ACE2) receptor for docking and entry and replicate efficiently in primary human airway epithelial cells. These data document the presence of a large animal reservoir of prepandemic SARS-like bat CoV that supports the possibility of direct bat-to-human transmission and recurrent outbreaks in the future [5,6,7].

In 2012, the antigenically distinct Middle East respiratory syndrome coronavirus (MERS-CoV) emerged and it is continuing to cause an ongoing outbreak in Saudi Arabia, the Arabian Peninsula and eastern Africa. Currently, 1626 infections in 26 countries have been reported with a mortality rate of ~30% [8]. Most of the cases are connected to the Arabian Peninsula, where camels, and perhaps bats, have been identified as the natural reservoir. While animal-to-human transmission has been responsible for a majority of cases, human-to-human transmission has also been described in hospital settings and in the home, most notably in May 2015 in South Korea, where a MERS-CoV-infected individual returned from Bahrain, causing more than 170 cases by human-to-human transmission [9]. 

Both pathogens cause severe lower respiratory tract infections, with the most severely impacted individuals developing acute respiratory distress syndrome (ARDS), a severe end-stage lung disease with poor treatment options and high fatality rates. Although asymptomatic infections were rare during the SARS-CoV epidemic, MERS-CoV infections frequently result in asymptomatic infections leading to asymptomatic spread. In general, the molecular mechanisms governing virus pathogenesis and disease severity remain understudied and vaccines and therapeutics are still not available [9,10].

**Figure 1:**
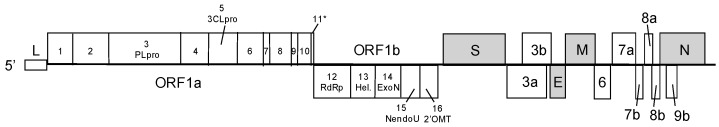
Genome organization of severe acute respiratory syndrome coronavirus (SARS-CoV). The SARS-CoV genome is approximately 29.7 kb and encodes for 14 open reading frames (ORFs). The 5′ end is capped and contains a leader sequence (L). SARS-CoV encodes for 14 ORFs, including ORF1, which is processed into nsp1 to nsp16, 4 structural ORFs (S, E, M, and N) in grey, and luxury downstream ORFs (3a, 3b, 6, 7a, 7b, 8a, 8b and 9b). nsp: nonstructural protein; S: spike; E: envelope; M: matrix; and N: nucleoprotein.

Coronaviruses are enveloped RNA viruses, containing the largest currently-known single-stranded, positive-sense RNA genome, which ranges in length from 25.5 to 32 kb. The viral particles range from 70 to 120 nm and are surrounded by ‘spike’-shaped glycoproteins, which give the viruses their characteristic ‘corona-like’ appearance in the electron microscope. Coronaviruses encode for 7 to 14 open reading frames (ORFs). ORF1 compromises approximately two thirds of the genome at the 5′-end and consists of two overlapping ORFs, ORF1a and ORF1b. Both ORFs are translated into large polyproteins, the precursor of at least 16 nonstructural proteins (nsps) that encode the viral replication machinery, and other important functions in virus–host interaction, like innate immune antagonism and pathogenesis. By ribosomal frameshifting, ORF1 is expressed as two polyproteins designated ORF1a and ORF2b, which are processed by a papain-like protease (PLpro) and the viral main protease (3CLpro), into at least 16 proteins. Unique among RNA viruses, coronaviruses encode a proof-reading complex, consisting of nsp10/nsp14, nsp12 (replicase) and perhaps nsp16, which regulates fidelity [11]. Downstream of ORF1, the genome also encodes for four structural proteins, the S (spike), E (envelope), M (matrix), and N (nucleoprotein) protein. These genes are interspersed with several additional luxury ORFs, which differ significantly among coronavirus in number, nucleotide sequence, gene order, and function; for SARS-CoV, most of these additional ORFs are indispensable for viral replication but many have been shown to antagonize the innate immune response and influence disease severity (Figure 1) [12]. 

## 2. Innate Immunity and Coronavirus Infections

Innate immunity is one of the earliest barriers to coronavirus infection. Following infection, pathogen recognition receptors (PRRs) such as retinoic acid-inducible gene I (RIG-I), melanoma differentiation-associated gene 5 (MDA5), Toll-like receptors (e.g., TLR 3, 4 and 7) and other sensing molecules recognize pathogen-associated molecular patterns (PAMPs) in viral components, such as viral structure proteins or viral nucleic acid. Successful recognition initiates a signaling cascade that activates an antiviral state in the host. Several main players of innate immunity, such as signal transducer and activator of transcription 1 (STAT1), myeloid differentiation primary response gene 88 (MyD88), TLR4, TLR7 and TLR3/TIR-domain-containing adapter-inducing interferon-β (TRIF), function to dampen infection severity during coronavirus infection in vivo [13,14,15,16,17]. Similarly, interferons (IFN−alpha (α) and IFN-beta (β), IFN-gamma (γ)) also play critical roles in controlling SARS-CoV in vivo and in vitro [12,13,14,15,16,18,19]. Data from the 2002/2003 outbreak have demonstrated that differential IFN and interferon-stimulated gene (ISG) expression levels in patients correlated with SARS disease outcomes. Several mouse models for SARS-CoV pathogenesis confirmed protective roles of MyD88, TLRs and select ISGs [12,14,16,20].

Like other viral pathogens, coronaviruses such as SARS-CoV and MERS-CoV have evolved genetic functions that delay and/or antagonize pathogen recognition as well as ISG effector functions. SARS-CoV encodes several proteins that modulate innate immune signaling through the antagonism of the induction of interferon. As mentioned above, several nsps that are encoded by ORF1 (ORF1a/b), like nsp1, nsp3 papain-like protease, nsp14 and nsp16 antagonize various sensing or signaling programs and NFκβ, or function to cap viral messenger RNAs (mRNAs) and evade interferon-induced protein with tetratricopeptide repeats (IFIT) 1-3 ISGs [11,21,22,23,24,25] (Figure 1). These nsps show high homology to proteins of other human coronaviruses and are critical for efficient viral replication. Several downstream open reading frames like ORF3a, ORF3b and ORF6 also antagonize sensing or signaling pathways, or block karyopherin 2 nuclear import [13,21] (Figure 1). MERS-CoV also encodes several luxury functions with interferon antagonism activities, including ORF4a, ORF4b and perhaps ORF5, noting that ORF4b antagonizes phosphodiesterase activity and RNAse L activation [26,27,28,29,30]. However, how the exact underlying mechanisms allow these antagonistic molecules to interfere with the effector molecules that establish an antiviral state, assist in wound repair, or prime and enhance an adaptive immune response, which is critical for clearance, is still under study. Recent studies have suggested that RNA viruses like coronaviruses and influenza viruses are able to manipulate the host’s epigenome, potentially heralding entirely new mechanisms of viral antagonism and new targets for therapeutic intervention and control [18,31]. The purpose of this review is to summarize the available methodology to study the epigenetic mechanisms that allow study of the epigenome during infection with coronavirus and other RNA viruses. 

## 3. Epigenetics

Epigenetic regulation bridges genotype and phenotype by changing the function of the gene locus without changing the sequence of the underlying DNA. Over the last decade, research efforts have revealed a dynamic range of epigenetic factors that shape and regulate chromatin status, leading to changes in host gene expression patterns, and therefore to alterations in phenotypes. Epigenetic modifications are significant in regulating cellular mechanisms and pathways during embryonic development, in memory function, in immunity and in disease [32,33]. While mutations directly affect the genetic material by changing the genetic code, epigenetic modifications change the chromatin structure or modify the nucleic acid without altering the genetic code. This makes epigenetic modifications reversible, flexible, and quickly responsive to changes in the environment and other exposures. Based on this ability, the study of epigenetic modifications is an important interface between the environment and the genome [32]. Over the last decade, epigenetics research has made rapid progress in understanding developmental biology, memory, and inheritability functions. More recently, it has become increasingly important in studies of oncology, adaptive and innate immunity, and infectious diseases [34,35]. It is becoming well established that many DNA viruses, and to some lesser extent RNA viruses, have evolved functions that antagonize the regulatory machine of the host epigenome, leading to regulated changes in host gene expression that lead to a favorable environment for virus replication and spread [36]. 

Over the last 20 years, the development of many biochemical and in particular high-throughput approaches have revolutionized our understanding of chromatin biology and function. Chromatin biology is now at the point where studies can be performed that use its tools to discover and validate new players and pathways in epigenetics and their role in a variety of biological disciplines, including developmental biology, oncology and infectious diseases, like bacterial and viral infections.

The human genome project (HGP) was officially completed in 2003, providing the research community with a detailed map of the genetic organization and structure of the human genome as well as the epigenome [37]. Another benefit of the human genome project was the development of next-generation sequencing (NGS) technology. Epigenetics research adopted the NGS techniques early on by refining methods like ChIP-Seq, RNA-Seq, and MeDIP-Seq [38,39]. Today, these are routine methods for the investigation of genome-wide changes in DNA methylation, histone modification, and DNA–protein interactions. Similarly, in the field of infectious disease research, high-throughput DNA analyses have enabled the genome-wide examination of epigenetic modifications and DNA methylation, providing systematic, large-scale association testing with disease phenotypes. It is likely that many common diseases, cancers, and infectious disease outcomes in humans are mediated by genetic and environmental factors. Likewise, epigenome-wide association studies (EWAS) provide a systematic identification of genome-wide epigenetic variants associated with disease outcomes [40]. EWAS can collect information about variation of epigenetic markers, global epigenetic patterns, and genome-wide distribution of epigenetic markers which can provide functional correlation with genotypes and phenotypes associated with particular pathological or non-pathological outcomes, defining new disease-associated marks [40,41]. In particular, the Encyclopedia of DNA Elements (ENCODE) project has advanced our understanding of the principles of genome, epigenome and chromatin organization, discovering and identifying formerly unknown histone modifications, nucleosome positions, and chromosome-wide maps of regulatory chromatin structures [38,42,43]. GENCODE, a part of ENCODE, now contains an extensive catalogued transcript, and pseudogene and long noncoding RNA (lncRNA) resources, helping to develop and to identify histone modifications and variants from several combinatorial patterns that define active promoters/TSSs (transcription start sites), transcribed gene bodies, inactive regions, and enhancers [44,45]. Several techniques described below are now commonly used in studies that integrate different data types including transcriptomics, proteomics, and epigenomics [46]. These allow us to validate and discover new molecular pathways that could lead to new discoveries in developmental biology, memory, and disease.

## 4. Chromatin

The genetic information in eukaryotic cells is encoded in the chromosomes and mitochondrial DNA. Chromosomes exist in deoxyribonucleoprotein complexes called chromatin. Chromatin is found in two variations: the euchromatin and the heterochromatin, which were originally distinguished cytogenetically by Giemsa staining procedures. Darker staining heterochromatin indicates tightly packaged protein and nucleic acid complexes found at centromers and telomers. These contain mostly inactive satellite DNA as opposed to the lighter-stained loosely-packed euchromatin, which defines genome regions under active transcription and gene expression [47]. 

Chromatin organization is complex and composed of a specialized set of proteins—the histones (H)—that organize the DNA into the nucleosome. The nucleosome is composed of a tightly-packed histone octamer consisting of the core histones H3, H4, H2A, and H2B with roughly 165 base pairs of DNA wrapped around it, much like beads on a string. This structure maintains stability and most importantly, protein and transcription factor accessibility to the DNA genome, allowing the chromatin to guarantee tight packaging of the genomic DNA, accurate replication, and distribution into the daughter cells during cell division, as well as transcriptional regulation of gene expression [48].

The histone’s N-termini, the so-called histone tails, extend from the globular protein unit and as such are targets for post-translational modifications. At this time, several chemical modifications have been identified and characterized: lysine acetylation, lysine and arginine methylation, serine and threonine phosphorylation, and lysine ubiquitination and sumoylation. These modifications are found on H2A, H2B, H3, and H4 histone subunits [48,49]. In 2001, Jenuwein and colleagues described the histone code, hypothesizing that a coding mechanism within the chromatin structure is regulated by chemical modifications to the histone tail, a concept which is now well supported by the literature [50]. We now know that distinct modifications of the histone tails interact with different sets of chromatin-associated proteins (Table 1). As a result, modifications on the same or different histone tails may be interdependent and generate various combinations on any one nucleosome, thereby supporting the modification-induced recruitment of chromatin-associated proteins. Consequently, the specificity of the downstream information is guaranteed and a specific crosstalk between histone modifications is possible.

The last decade has shown that this regulatory instance of nucleosomes and chromatin structure on the genome has emerged as a critically important determinant of cellular transcription, replication, and differentiation state.

## 5. Epigenetic Regulation of Gene Expression

### 5.1. Histone Modifications and Transcription

**Figure 2:**
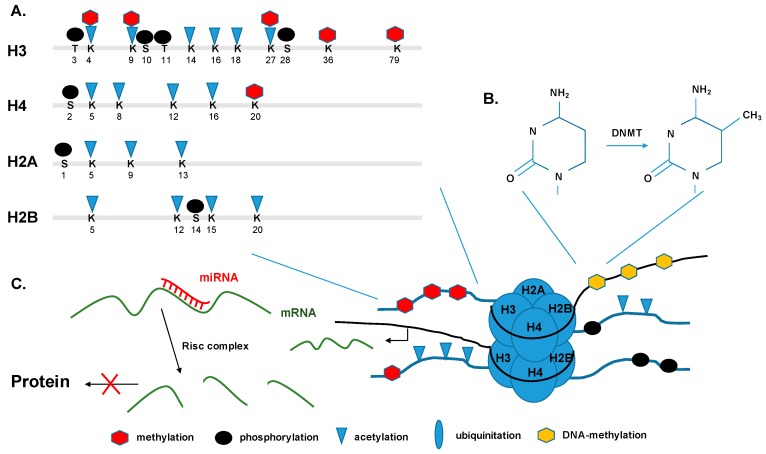
Epigenetic modifications. (**A**) Histone tails are targets for post-translational covalent modification. Particular lysine residues (K) can be methylated, acetylated, phosphorylated, and ubiquitinated, and particular serine residues (S) can be also phosphorylated. (**B**) DNA is being methylated by transferring a methyl group to the C-5 position on cytosine bases. (**C**) miRNA, usually 22nt long, bind the 3′ end of their target mRNA with their seed region and mediate the degradation of the mRNA by incorporation into RISC. DNMT: DNA methyltransferase; mRNA: messenger RNA; miRNA: microRNA; RISC: RNA-induced silencing complex; K: lysine; S: serine; T: threonine.

The histone tails of the nucleosome are subject to post-translational modulations. These modulations are covalently attached to the tails and include methylation of arginine residues, and methylation, acetylation, ubiquitination, phosphorylation, and sumoylation of serines (S) and threonines (T) (Table 1, Figure 2A). 

Over the last decade, many modifications have been associated with active or non-active transcription. Modifications like the acetylation (ac) of H3 and H4, as well as the di- or tri-methylation (me) of H3 on lysine 4 (H3K4), are associated with an active transcription state. On the other hand, methylation of H3K9 and H3K27 are now associated with transcriptional repression of the particular gene [52]. Based on their function within gene expression the particular histone marks can be found in distinct localizations within a gene region [53].

Likewise, many modifications are almost uniquely associated with gene organization components, like promoters, TSSs, enhancers or gene bodies, slicing sites, and transcriptional end sites (TES). These histone marks help to organize the chromatin by modulating accessibility, thereby defining regulatory regions and elements, like promoters, enhancers and insulators, within the genome [54]. 

Some of the histone mark distributions are uniquely associated with the transcription rates of particular genes. As mentioned above, main histone marks for regulating the TSS are H3K4me3 and H3K27me3; both modifications are exclusively found at the TSS and in the appropriate promoter region of the particular gene [55]. H3K4me3 is the main modification for an active promoter region and therefore actively transcribed chromatin, while H3K27me3 on the other hand, is the main modification found at repressed promoters [56]. 

Despite these differences, both modifications perform a crucial function in bivalent or ‘poised’ promoters [53]. A promoter occupied with both H3K4me3 and H3K27me3 can be rapidly activated or inactivated for transcription, making both histone modifications signature configurations for poising bivalent promoters for alternate fates: active and repressed gene transcription [51,57,58]. Other histone marks are preferentially located in enhancer regions or within the gene body. For example, H3K27ac and H3K4me1 are enriched at active enhancer sequences, active promoters are flanked by H3K27ac and H3K4me3, and gene bodies show enrichment of H3K36me3. 

The state of the chromatin is modulated by a large number of proteins which can be seen as ‘writers’, ‘readers’, and ‘erasers’ [59]. ‘Writers’ are responsible for encrypting the information capacity of nucleosomes by adding distinct post-translational modifications to the histone tails. Generally, ‘writers’ are acetylases, methylases, and phosphorylases that specifically add the appropriate modification to the histone tails. ‘Erasers’ antagonize the function of the ‘writers’, and remove the histone modifications. These enzymes include deacetylases, demethylases, and phosphatases. ‘Writers’ and ‘erasers’ modulate the assembly, placement, recognition, and modification. 

The recognition of histone modifications is mediated by ‘readers’; proteins which are tightly regulated by phosphorylation and dephosphorylation through signaling pathways, recruitment and binding of co-factors, like transcription factors and adaptor proteins [59]. Overall, histone modification patterns are dynamic and reflect the activation state of a gene, the elongation state and the splicing patterns of the pre-mRNA transcript. To match the complexity of the modification patterns, ‘readers’ are often organized in protein complexes, containing a bromodomain, chromodomain, and tudor domains and harboring several putative modification-dependent binding sites. Many of the histone modifying enzymes belong to complex protein superfamilies that show stringent substrate, catalytic, and tissue specificity. This way, these proteins regulate DNA accessibility together with ATP-dependent chromatin remodeling complexes, which are mediating remodeling of nucleosomes, like moving, ejecting or restructuring nucleosomes. This mediates pioneer transcription factors, that are involved in recruiting transcription machinery complexes, like the RNA polymerase II (Pol II) complex, or insulator proteins, to bind at nucleosome-free DNA regions and initiate gene expression. This guarantees a specific modification and response to environmental stimuli [60].

Based on the theory of the histone code, the same histone mark can have very different physiological outcomes depending on the location in the chromatin, the neighboring modifications, and the combination of modifications [50]. In other words, to understand the function of a single histone mark, the combination and the co-occurrence with other marks needs be considered. This complexity determines that a combination of multiple histone modifications can have a cascading effect with a variety of different outcomes ranging from transcription repression to transcription activation to transcription termination. These varying outcomes regulate combinatory and sequentially downstream functions, generating distinct signatures for every individual gene [50,61].

This circumstance influences strongly the nature of the modification-binding proteins, the so-called readers and the following down-stream processes. This way, a rather small set of separate histone modifications results in a broad range of different outcomes for the cell. Overall, it is not surprising that these chromatin-modifying enzymes play an important role in maintaining chromatin structure and dynamics. It is important to note, however, that chromatin marks can be easily reversed. As a consequence, they can rapidly respond to external stimuli, thereby regulating the accessibility of the underlying DNA to the transcriptional machinery and ensuring the correct association of expressed genes in the appropriate situation [62,63]. 

### 5.2. DNA Methylation and De-Novo Methylation

In all mammalian cells, DNA methylation takes place post DNA replication. It occurs at the 5′ position of the cytosine ring within CpG nucleotides by adding a methyl group to create 5-methylcytosine (5mC). The modification is mediated by a family of enzymes, the DNA methyltransferases (DNMTs) (Figure 2B). DNMT3a and DNMT3b have been described as de novo methyltransferases, preferentially targeting unmethylated CpG islands (CGIs) in the genome to initiate DNA methylation [64]. Studies have shown that DNMT1 functions as maintenance methyltrasferase, ensuring that the methylation status is maintained during DNA replication and following cell division [65]. However, DNA methylation as an epigenetic marker is highly dynamic, and therefore crucial in gene silencing and gene regulation, the establishment of heterochromatin, and in regulating the stability of the chromosome [66]. Hypermethylation of repetitive DNA sequences in combination with certain histone marks results in the condensation of chromatin and therefore in the establishment of heterochromatin [67]. Recently, DNA hydroxymethylation (5hmC) has been identified as another form of DNA methylation. Several studies have shown that enzymes of the ten-eleven translocation (TET)-family catalyze the modification and that it has a major role in embryonic neuronal development. [68,69].

The presence of 5mC CGIs plays a critical role in regulation of gene expression. More than 60% of coding genes contain CGIs in promoter-associated regions. These CGIs are generally unmethylated, and therefore easily accessible to transcription factors and other chromatin-associated proteins for the expression of most housekeeping genes and other regulated genes [67]. However, de novo methylation of those promoter-associated CGIs will repress and silence promoter activity. Transcriptional inactivity at a methylated promoter region can be reversed by methyltransferases, rendering the DNA sequence into active chromatin, therefore demonstrating another instance of gene regulation [51,64,67,70].

### 5.3. Non-Coding RNAs and micro RNAs

Starting in the early 2000s, the field of non-coding RNAs (ncRNAs) evolved from its historic origins as “junk RNA” and quickly expanded into its own field of research. Based on their function and their genetic origin ncRNAs can be divided into long non-coding RNAs (lncRNAs) and small non-coding RNAs (sncRNAs), based on whether each RNA is greater than or less than 200 bp in length [71]. For many years, lncRNAs were considered to be unimportant junk byproducts of evolution and were ignored by most of the research community. However, this group of RNAs is now recognized as a critical regulator in chromatin remodeling, transcriptional regulation, and post-transcriptional processing [72]. Epigenetics and microRNAs (miRNAs) regulate whole gene expression patterns transcriptionally and post-transcriptionally, respectively (Figure 2C) [73]. At the same time, epigenetics and miRNAs control each other to form a regulatory circuit and to maintain normal physiological functions [74]. Several miRNAs have been identified that target genes that control epigenetic pathways, like DNMTs and histone methyltransferases (HMTs), thus controlling chromatin structure by regulating by regulating histone modifier molecules. The expression of miRNAs on the other hand is regulated by histone modification and DNA methylation, forming an epigenetics–miRNA regulatory circuit [73].

## 6. Methods to Study Epigenetics

**Figure 3:**
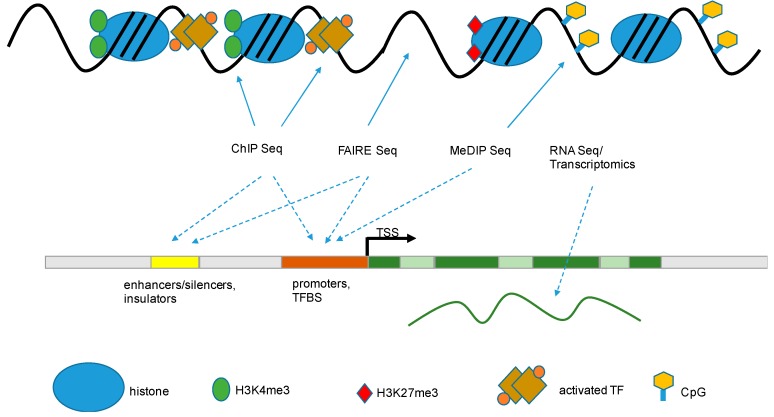
Methods of epigenetic analysis. Schematic representation of the major epigenetic methods that are being used to detect epigenetic modifications (solid arrow 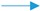) that are associated with functional gene components (dashed arrow 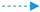), like promoters, transcriptional start sites, enhancers, gene bodies, slicing sites, and transcriptional end sites [43]. TSS: transcription start site; TF: transcription factor; TFBS: transcription factor binding site; FAIRE: formaldehyde-assisted isolation of regulatory elements; ChIP: chromatin immuno-precipitation; MeDIP: methylated DNA immunoprecipitation.

A number of high throughput technologies have been developed to study the epigenetic landscape and epigenetic modifications genome-wide and on sequence-specific levels (Figure 3). 

### 6.1. Modifications of Histones and Localizations of Histone Marks within the Genome

#### 6.1.1. Formaldehyde-Assisted Isolation of Regulatory Elements (FAIRE)

As a broad strategy to identify modifications of histones and the localization of histone marks across the genome, FAIRE was developed and has been applied to understand the chromatin status of target cells and of DNA viruses under different conditions of infection [75]. FAIRE is a method to isolate regulatory elements from eukaryote chromatin, thereby taking advantage of the fact that DNA segments that actively regulate transcription in vivo are typically characterized by eviction of nucleosomes. The FAIRE method involves crosslinking the chromatin by adding formaldehyde, which preferentially targets heavily-condensed, transcriptionally-repressed chromatin over transcriptionally-active chromatin. The crosslinked chromatin is then sheared by sonication; phenol-chloroform is added to separate protein (nucleosome-depleted) DNA fragments from nucleosome-covered DNA. Downstream detection methods include microarrays, NGS, or quantitative PCR. The regions isolated and detected by FAIRE are largely coincident with the location of open chromatin, such as DNase hypersensitive sites, TSS, enhancers, and actively-transcribed promoters [51,76,77,78].

#### 6.1.2. Chromatin Immuno-Precipitation (ChIP) 

This technique is used to determine whether a given protein binds to, or is localized to, a specific DNA sequence in vivo. Cross-linked chromatin is sheared and the DNA-binding protein of interest is precipitated by using a protein-specific antibody. The bound DNA is then isolated by reverting the cross-linking and can be analyzed by utilizing microarrays (ChIP-on-chip), next-generation sequencing (ChIP-Seq), and quantitative PCR (ChIP-PCR) [79,80]. The method is strictly dependent on the availability of high quality antibodies to the target protein. The availability of antibodies and the quality of the antibody used in the ChIP determines the quality of the data generated by the study. In general, only antibodies with high sensitivity and specificity should be considered for use, because this will allow the detection of enrichment peaks without substantial background noise [81].

The combination of the ChIP technology with next-generation sequencing allows and improves the characterization of binding sites for transcription factors and other DNA-binding proteins and the identification and characterization of DNA sequence motifs across the entire genome. The advancement in high resolution is crucial in profiling nucleosome positioning, the systematic cataloguing of histone modification patterns, and the establishment of precise histone modification maps throughout the entire genome [51,80,82]. 

### 6.2. Whole-Genome Methylation Status

To quantify the global distribution of active and inactive states of chromatin across the genome, several methods and technologies have been developed to measure the methylation status across the genomic DNA [83]. Recently, the methylated DNA immunoprecipitation (MeDIP) technique was developed and has proven to be a versatile, unbiased approach to study the methylation status of either the whole genome or specific regions of interest. In brief, genomic DNA is sheared and precipitated with a monoclonal antibody that recognizes 5-methylcytidine. Another approach based on immune precipitation is the Methyl-DNA binding protein ChIP using the Methyl-CpG-binding domain protein 2 (MBD2), a member of the MBD protein family. The resulting enrichment of methylated DNA can be determined by PCR to assess the methylation state of CpG islands in individual promoters or gene regions of interest. Alternatively, precipitated methylated DNA can be combined with large-scale analysis using microarrays or next-generation-sequencing [84,85].

There are other complementary approaches to study the genome-wide methylation status of chromatin based on methylation arrays and methylation-sensitive and methylation-insensitive restriction enzymes. The methylation array technology is based on the Infinium MethylationEPIC technology (formerly the Infinium Human Methylation 450 array) which allows low sample input and fast read-out but has the disadvantage of not covering all annotated genes and shows bias which is based on the array technology. Another approach uses restriction enzymes, like *Hpa*II/*Msp*I, which are blocked or not blocked by CpG methylation. After treatment of the total DNA with the enzymes the distribution and extent of DNA methylation can be analyzed by quantitative PCR targeting regions of interest. However, the enzymatic approach is prone to bias based on the sequence specificity of the utilized restriction enzymes. This limits the analysis to certain sequence 3 motifs, which can be unevenly distributed across the whole genome [86]. 

## 7. Immune System and Genetics

Based on functional and spatial patterns, the immune system is broadly divided into two broad arms: the innate immune system and the adaptive immune system. Both systems include a wide range of cell types that communicate via direct cell–cell interactions or by the secretion of mediators such as interleukins, cytokines, and chemokines. The innate immune system not only regulates cell intrinsic defense programs in response to microbial attack but also has a critical role in activating and shaping the adaptive immune response. The innate immune system accomplishes this by being able to generate and drive a transcriptional response that is both cell- and stimulus-specific. Based on these mechanisms, the signal-specific induced response guarantees initiation of the appropriate innate and adaptive immune responses that have the greatest potential to successfully control a particular pathogen [87,88,89]. 

Much of the innate immune response is regulated by membrane-bound and intracellular PAMPs, like TLRs, RIG-I, MDA-5 and cyclic GMP-AMP synthase (cGAS)- stimulator of interferon genes (STING) and other sensors that detect invading pathogens [90,91]. These PAMPs use unique and overlapping signaling cascades to activate effector transcriptional programs that regulate antimicrobial defense pathways. Most of the research has focused on elucidating the exact signaling programs that regulate antimicrobial defense to different pathogens and the microbial countermeasures that inactive specific pathways [92,93]. More recently, a growing body of evidence has determined that chromatin modifications and epigenetic regulation play a crucial role in shaping the activated host response to a microbial invasion [35,94,95]. Advances in sequencing technologies have significantly increased our ability to sensitively and specifically measure the transcriptional state at a single-cell level. Systems biology approaches have revealed the more complex gene interaction networks that become activated or repressed. These mechanisms have been essential in understanding the functional specialization of cells as individual units of the innate immune system, the flexibility in mounting innate and inflammatory immune response, and in deciphering the mechanism of communication and interactions within specific cell populations.

## 8. Epigenetic Regulation/Modulation of Host Response

A basic feature of innate immune cells is the ability to start a transcriptional response program that is specific to the stimulus, and then mounting a signal with a high degree of cell type and stimulus specificity [96,97,98].

Recent studies have involved epigenetic factors in every aspect of activation and shaping innate and adaptive immune responses. Major contributions are the:

➔ Recruitment of transcription factors/machinery;
➔ Prevention of unwanted expression of potent mediators; and
➔ Repression or activation of secondary gene programs [98,99].

The main players of the innate immune system are primary response genes like IFN and tumor necrosis factor (TNF), which are rapidly induced and whose promoters show the characteristics of a poised promoter. Often, the promoters of these genes also contain CpG islands which are resistant to epigenetic modifications like DNA methylation and histone tail modification. These common modifications can be found at promoters of highly active transcribed genes, which also show high levels of RNA Pol II occupancy [99]. To the contrary, ISGs usually display low levels of activating histone marks like H3K4me3, H4Ac, and low level RNA Pol II occupancy [100]. These genes often require additional transcription factors and chromatin remodelers, like recruitment of the ATP-dependent chromatin remodeling complex SWItch/sucrose non-fermentable (SWI/SNF) to initiate transcription [36,97]. 

Two cell types of the innate immune system, dendritic cells and macrophages, are the primary sensors of ‘danger’ signals. Once these cells are activated, it is especially important that their signals are both cell-specific and stimulus-specific to ensure the initiation of a temporal and spatial response. These cell-specific signals can be mediated through cell–cell contact or by secretion of IFN and TNF. Thus, the ability of their epigenome to change within minutes after a stimulus is not just essential for initiating a rapid antiviral host response but is also essential to ensure a persistent and specific defense response. This way, epigenetic mechanisms are responsible for the priming and the memory of these responses and for guaranteeing a functional and highly regulated host response beyond the initial activation wave.

Besides the histone marks for poised promoters H3K4me3 and H3K27me3, an important role as a major player in regulating the activation of IFN has been described for H3K9me2. Fang et al. correlated the levels of H3K9me2 modification with the level of interferon expression in vitro. H3K9me2 is a repressive histone mark that contributes to DNA methylation and heterochromatin formation and thereby prohibits histone tail acetylation by recruiting the transcriptional repressor of the heterochromatin protein 1 family [101]. However, in the above study, Fang et al. were able to demonstrate that the overall levels of H3K9me2 mark in the promoter region of the type I interferon and the expression of ISGs inversely correlates in dendritic cells, defining this histone modification as an important regulator of the IFN response [101,102].

On the other hand, H3K4me3 is a histone modification exclusively found at active promoters and is therefore often enriched in promoter regions regulating TLRs. A recent study has shown that 60 min after lipopolysaccharide (LPS) stimulation of macrophages and dendritic cells, the overall histone acetylation and the binding of polymerase II (Pol II) at the specific promoters was tremendously increased, demonstrating an efficient and specific induction of the innate immune response by epigenetic control mechanisms [103]. 

## 9. Coronaviruses/Influenza Viruses: Viral Antagonism of Host Gene Expression by Altering Histone Modifications

Interferons are important mediators of an antiviral state and initiators of pathogen-driven immune response by the inactivation of ISGs [104,105]. Therefore, it is likely that many viruses have evolved antagonistic mechanisms to overcome specific ISG effectors [106]. As discussed earlier, IFN and innate immune responses are subject to extensive epigenetic regulation, mediated by specific epigenetic marks, and the manipulation of histone modification enzymes, DNA methylases, and chromatin remodeling complexes. Viruses have evolved mechanisms to disturb and antagonize these epigenetic regulatory programs by (1) interfering with the host’s histone modification enzymes [107]; (2) interfering with the host’s chromatin remodeling machinery [108]; and (3) encoding for viral proteins that interact directly with the host’s modified histones [98,109]. 

Marazzi et al. have demonstrated that the highly pathogenic H3N2 influenza A virus inhibits the initiation of the host innate immune response in part by interfering with the epigenetic control of gene expression. Using histone mimicry, it has been proposed that the carboxyterminus of the H3N2 nonstructural protein NS1 shares homologue sequences with the aminoterminus of the histone H3 tail [31]. Essentially, the viral NS1 protein mimics the histone tail of the H3 histone and thereby interacts with the transcription complex, which usually docks to the H3K4 mark to initiate transcription [31,110]. 

Previously, we compared ISG profiles of pathogenic influenza viruses and coronaviruses in Calu3 cells, a human airway epithelial cell line, by using a transcriptomics and proteomics dataset [18]. The infection of Calu3 cells with the tested respiratory viruses resulted in diverse virus-specific ISG expression signatures. The highly pathogenic H5N1 avian influenza A (HPIA) virus showed a rapid manipulation of ISG with strong down- and up-regulation of specific ISG sets at 7 h post infection. In contrast, the 2009 pandemic H1N1 strain showed no ISG modulation, and the infected cells mounted a robust IFN-induced antiviral state starting at 3 h post infection. SARS-CoV infection of Calu3 cells also showed a strong induction of ISG effectors, but the response was significantly delayed with peak expression at 24 to 48 h post infection. In 2012, the newly emerged MERS-CoV showed delayed ISG production with effects visible at 18-h post infection (hpi), with significant inhibition of expression of specific ISG subsets. Overall, the viral manipulation of the host antiviral IFN response results in successful virus infection and viral replication, defining a viral antagonistic approach, which may be a mechanism to interfere with the host’s innate immune response [22]. 

**Figure 4:**
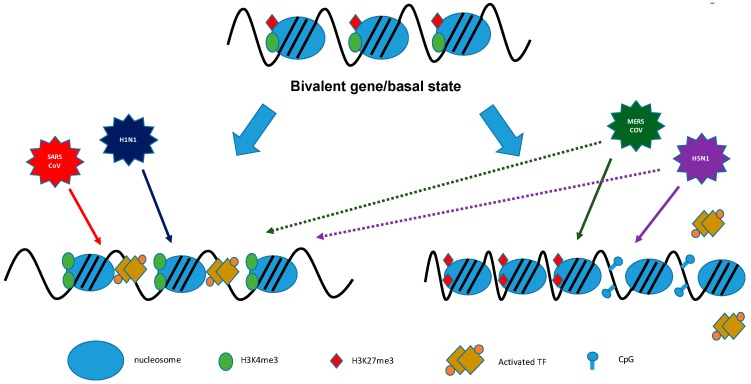
Epigenetic modification infection with highly virulent respiratory viruses. Infection with pathogenic influenza viruses and coronaviruses induces changes in the basal state of host chromatin. Infection with H1N1-09 (blue) and SARS-CoV (red) results in enrichment of H3K4me3 incorporation (green ovals) and depletion of H3K27 (red diamonds), and therefore in open, transcription-active chromatin. In contrast, H5N1-VN1203 and Middle East respiratory syndrome coronavirus (MERS-CoV) infection drives H3K27me3 enrichment and depletes H3K4me3 for a subset of genes, favoring a closed chromatin conformation that inhibits interferon-stimulated gene (ISG) expression [18].

In our laboratory, by using ChIP-PCR approaches, we could determine differential occupancy of histone marks at the promoters of ISG genes. We showed that the promoter regions of ISG genes contained more histones with active marks of H3K4me than the repressive H3K27me3 mark, therefore favoring open chromatin and promoting active transcription and ISG expression during H1N1-2009 and SARS-CoV infection. In contrast, infection of Calu3 with the highly pathogenic HPAI and MERS-CoV resulted in increased levels of H3K27me3 and decreased levels of H3K4me3 occupancy at the promoter regions of subsets of specific ISGs, which were not induced, demonstrating that these viruses have developed antagonistic mechanisms to specifically target the IFN arm of the innate immunity (Figure 4). 

**Figure 5:**
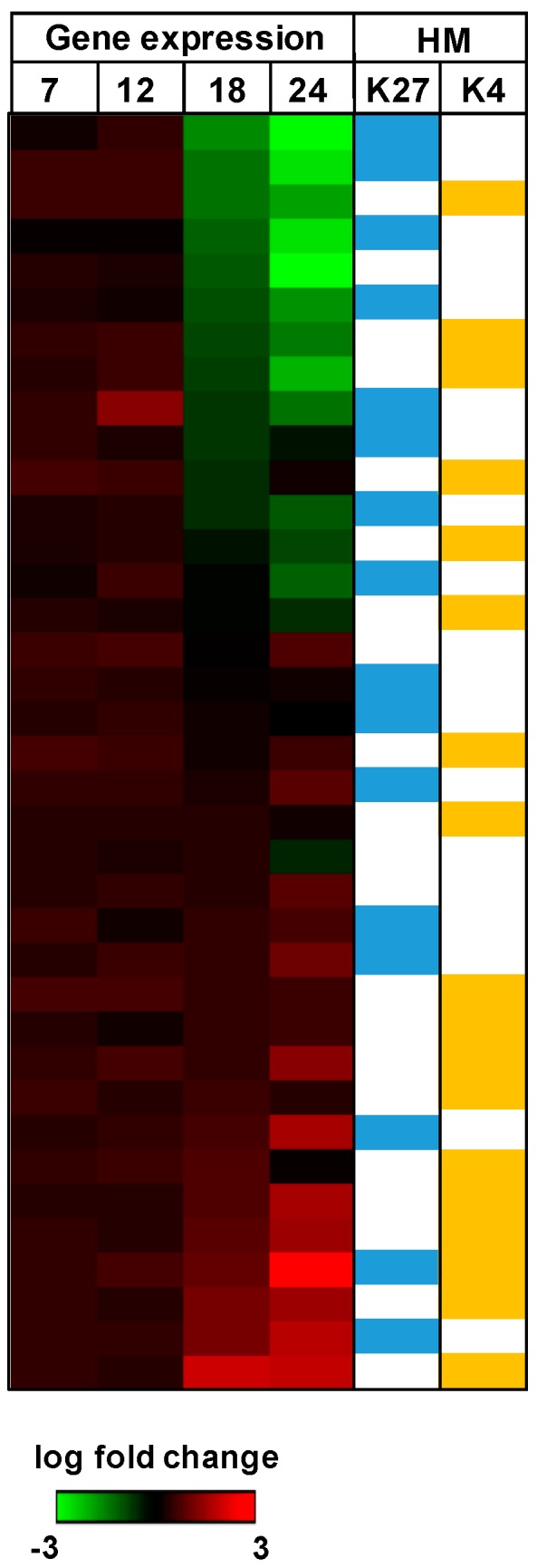
Interferon-stimulated gene (ISG) profile during MERS-CoV. Global ISG transcriptional response to MERS-CoV infection. Genes are ordered by 18 h post infection (hpi) expression levels [18]. Columns labelled HM (histone modification) indicate occupancy with H3K27me3 (K27, blue) and H3K4me3 (K4, yellow), respectively.

We have expanded this dataset by using a genome-wide ChIP-Seq approach. This allows us to choose any set of cellular effector molecules and to study their histone modification profile during infection. Figure 5 shows as an example the expression profile of ISGs during the early phase MERS-CoV infection. As already described by Menachery et al., expression of ISGs effectors occurred rather late after infection at 12 hpi to 18 hpi. However, MERS-CoV infection in Calu3 induces the up-regulation and down-regulation of ISGs. We then applied ChIP-Seq data to the expression data. When we looked at the expression level of ISG transcripts at 18 hpi, we could see how the histone modification promoter profile at 12 hpi corresponded with the transcriptomic data. ISGs that were downregulated during infection showed increased occupancy of H3K27m3 modification in their promoter region (indicated in yellow) and ISGs that were upregulated during infection showed increased occupancy of H3K4me3 within their promoter region (blue).

While histone mimicry has been identified for H3N2 virus, the responsible binding motif is not contained in NS1 protein encoded by H5N1-VN1203, although the NS1 protein may contribute to this phenotype [31]. This suggests that different NS1 proteins may mediate the downregulation of subsets of ISGs by different mechanisms depending on Influenza Type A virus (IAV) strain. For H5N1-VN1203, NS1 may inhibit ISG expression by mimicking different histones, targeting histone-modifying enzymes, or disrupting a histone adaptor protein complex [18]. Several IFN antagonists have been identified; however, the nature of the accessory protein mediating ISG downregulation by interfering with the host’s epigenome remains to be identified [18,111].

## 10. Systems-Biology Approach

### 10.1. A Model Platform for Epigenetic Research Following Coronaviruses and Other Respiratory Virus Infections

**Figure 6:**
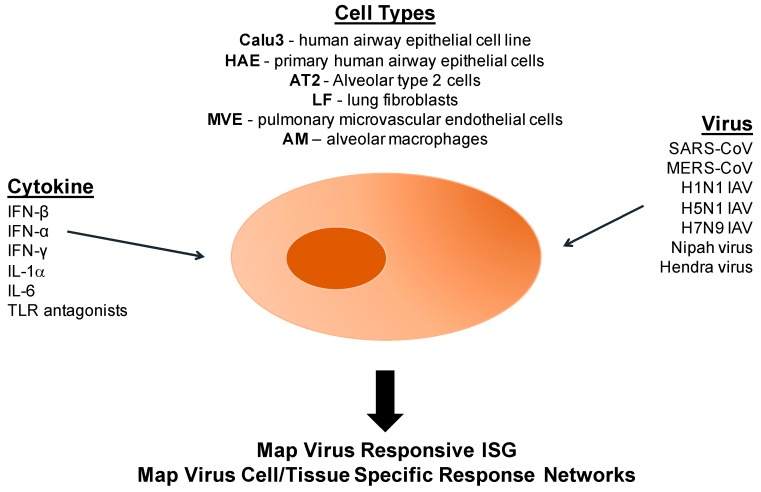
Model system for the study of coronaviruses and other respiratory viruses. Human respiratory cell lines (Calu3) or primary cells (HAE, AT2, LF, MEV, and AM) are used as a minimalistic model system to study the response to perturbations like cytokine treatment and virus infection. IFN: interferon; TLR: Toll-like receptors; IL: interleukin; IAV: Influenza Type A virus; CoV: coronavirus.

Calu3 cells are a continuous human airway epithelial cell line that can be differentiated in ciliated cells and are commonly used to study respiratory cell function under different physiological stresses and conditions [112]. By utilizing Calu3 cells, we have developed a robust human model platform to study innate immune regulatory control and epigenetics following emerging coronavirus and influenza virus infections as well as other highly pathogenic viruses (Figure 6). The first step is to define expression changes following treatment with defined perturbations, like various cytokines, to identify smaller subsets of effector gene expression patterns (RNA and protein) that are downstream of a specific signaling pathway. In parallel, the Calu3 cells are then infected with different highly pathogenic emerging or contemporary respiratory viruses and global proteomic and transcriptomic expression patterns characterized at different times post infection (Figure 6). By filtering expression changes to specific subsets of cytokine specific gene sets after infection, novel patterns of virus-induced regulatory control are revealed while identifying novel gene sets for downstream epigenetic and virus studies. For example, the same set of ISGs are either globally induced rapidly or differentially induced following H1N1 and H5N1 infection. In contrast, ISG expression patterns are significantly delayed but either globally or differentially induced following SARS-CoV and MERS-CoV infection, respectively [18]. Expression pattern differences were independent of any specific transcription factor function, but rather were regulated primarily by epigenetic control mechanisms. Importantly, these studies seed future studies using other viruses and/or segwaying into primary human airway epithelial cells (HAE), primary type II pneumocytes (AT2), lung fibroblast (LF), pulmonary endothelial cells (MEV) and resident immune cell populations in the lung [18,113]. Utilizing these model systems, we aim to study genome-wide histone modifications, DNA methylation patterns, and the chromatin landscape after virus infection across different cell types in the lung, revealing cell type-specific regulatory features that function to regulate infection outcomes. The goals of recently performed studies in our laboratory sets the stage to determine if host epigenetic processes play a crucial role in controlling transcriptional regulatory networks that antagonize or promote MERS-CoV and H5N1 infection and pathogenesis. While the molecular mechanisms regulating epigenetic control remain elusive following emerging CoV infection, a growing body of evidence suggests that viruses, even RNA viruses that replicate in the cytoplasm, interfere with the host’s epigenome.

### 10.2. Integration across Data Types

Host–pathogen relationships are plastic and dynamic. In particular, selective pressure put on the pathogen by the host increases the degree of plasticity and adaptability of the pathogen. On the other hand, the host phenotype is altered by the pathogen and the pathogen’s virulence, which leads to a co-adaptation that influences pathogen and host equally. For a successful viral life cycle, viruses have co-evolved with the host, which often means that the virus has to adjust to the host’s immune system, resulting in distinct mechanisms to repress or evade the host’s immune response. Co-evolution with the host may well have expanded the capabilities of epigenetic regulation, adding additional mechanisms and sources of dynamic reversible phenotype variation [109]. The evolution of viral antagonistic mechanisms which interfere with the host’s gene expression ability by modifying histone marks and therefore chromatin dynamics, enabling viruses to attack entire immune gene clusters, has not been heavily investigated and may represent a productive arena for future study. Mechanisms by which viruses could modulate the host’s chromatin include sequestration and displacement of chromatin-associated proteins, interference with the chromatin remodeling machinery, DNA-binding transcription factors, histone modifying enzymes, and direct alteration of methylation and acetylation state of histones and histone mimicry [18,31,114,115].

**Figure 7:**
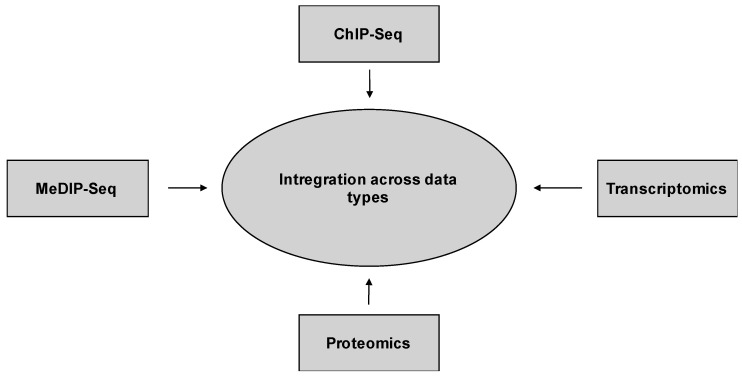
Integration across data types. Epigenetic approaches combined with transcriptomics and proteomics datasets provide a spatial-temporal data integration approach to identify regulatory genomic clusters and regions playing a role in viral infection.

Several complementary approaches can be combined to study, define, and map the epigenetic landscape during coronavirus infection and other highly pathogenic human viruses (Figure 6). Combining systems biology approaches like epigenomic, transcriptomic and proteomic datasets provides a data integration approach to identify regulatory genomic clusters and regions that play a crucial role in the host’s antiviral response (Figure 7) [116,117]. Moreover, systems biology approaches allow the development of models to make comparisons of data across pathogens to better predict complex biological systems. For example, the integration across systems-based data types will allow us to predict gene expression and to infer new gene regulatory networks. Using comparatively generated data from multiple levels of biological systems will allow the association between phenotypic outcome and variation, and the prediction of gene expression using only a few epigenetic features (Figure 7) [117,118]. These genome-wide based predictions are essential to define and interpret gene regulatory networks (GRNs). By linking epigenetic features to gene activation, gene expression levels are integrated into enrichment profiles which can be interpreted as clusters of differential enrichment patterns and then used to map interactions of virus and host to infer the identity of genes that are implicated in disease and pathogenesis associated with the virus [117].

## 11. Outlook

Recent advancements through basic research in the understanding of the mechanisms involved in viral chromatin modification in lytic viruses have opened a new window into previously unknown mechanisms of viral antagonism and host–virus interactions, including genetic factors that influence both protective or pathogenic host responses. The subsequent identification and improved understanding of these mechanisms open new avenues for the development of antiviral drugs by illuminating new targets for specific inhibitors. Established model systems for latent and persistent viruses such as human immunodeficiency virus 1 (HIV-1) and herpesviruses have already demonstrated modulation of the viral infection by chromatin [119,120]. As described here, a growing number of studies also show modulation of viral infection by chromatin in lytic virus infections. The emerging parallels between the existing knowledge of chromatin’s effect on and interaction with latent and persistent viruses and the emerging understanding of its comparable interaction with lytic viruses suggest that a greater focus on chromatin-based therapies for a variety of virus families could reveal fundamental new landscapes of virus–host interaction that play critical roles in disease severity.

## Figures and Tables

**Table 1:** Histone (H) modification and their role in transcription (list is not complete, only well-established motifs in the literature are listed) [49,51].

Modification	Role in Transcription	Modification Site
Acetylation	Activation	H3ac, H3K9ac, H3K14ac, H3K27ac
Methylation	Activation	H3K4me1, H3K4me2, H3K4me3, H3K36me3, H3K79me2
Methylation	Repression	H3K9me3, H3K27me3
Phosphorylation	Activation	H3S10
